# A Toroidal Zr_70_ Oxysulfate Cluster and Its Diverse Packing Structures

**DOI:** 10.1002/anie.202010847

**Published:** 2020-11-11

**Authors:** Sigurd Øien‐Ødegaard, Calliope Bazioti, Evgeniy A. Redekop, Øystein Prytz, Karl Petter Lillerud, Unni Olsbye

**Affiliations:** ^1^ Centre for Materials Science and Nanotechnology Department of Chemistry University of Oslo P.O. Box 1033 Blindern N-0315 Oslo Norway; ^2^ Centre for Materials Science and Nanotechnology Department of Physics University of Oslo P.O. Box 1048 Blindern N-0316 Oslo Norway

**Keywords:** microporous materials, self-assembly, supramolecular chemistry, zirconium

## Abstract

Herein, we report the discovery of a toroidal inorganic cluster of zirconium(IV) oxysulfate of unprecedented size with the formula Zr_70_(SO_4_)_58_(O/OH)_146_⋅*x*(H_2_O) (Zr_70_), which displays different packing of ring units and thus several polymorphic crystal structures. The ring measures over 3 nm across, has an inner cavity of 1 nm and displays a pseudo‐10‐fold rotational symmetry of Zr_6_ octahedra bridged by an additional Zr in the outer rim of the ring. Depending on the co‐crystallizing species, the rings form various crystalline phases in which the torus units are connected in extended chain and network structures. One phase, in which the ring units are arranged in layers and form one‐dimensional channels, displays high permanent porosity (BET surface area: 241 m^2^ g^−1^), and thus demonstrates a functional property for potential use in, for example, adsorption or heterogeneous catalysis.

## Graphical abstract

A toroidal Zr(IV) oxysulfate cluster of unprecedented size with the formula Zr_70_(SO_4_)_58_(O/OH)_146_⋅*x* (H_2_O) (Zr_70_) was synthesized. Depending on the co‐crystallizing species, it displays different packing of the ring units. One of the phases contains one‐dimensional channels through the rings, showing high permanent porosity (surface area: 241 m^2^ g^−1^). The ring entity measures over 3 nm across and displays a pseudo‐10‐fold rotational symmetry.

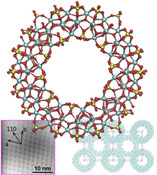

Toroidal (ring‐shaped) units of molecular dimensions are visually appealing due to their peculiar symmetry, have an intrinsic high surface area due to their geometry and the possibility of forming host‐guest ensembles by filling of their inner cavity. These traits are favorable for the formation of regular, yet multifunctional surfaces, which can be employed in for example, adsorption and catalysis. Ring‐shaped inorganic entities on the nanometer scale, albeit rare, are normally found as (ionic) polyoxometalates (POMs) and coordination complexes (containing organic ligands), prominent examples of which include the Mo_154_ “big‐wheel”,^[1]^ Pd macrocycles discovered by directed solution techniques,^[2]^ and more recently a gigantic molecular wheel of Gd_140_ ^[3]^ and a U_70_ unit.^[4]^

Zr‐based clusters have gained considerable attention after the discovery of carboxylate oxoclusters and, especially, Zr‐based Metal‐Organic Frameworks (MOFs). The hexameric dodecacarboxylate cluster found in most Zr‐based MOFs was first isolated from an organic solution in 1997,^[5]^ then as a glycine cluster from aqueous phase (coincidently with non‐coordinated sulfate) in 2008,^[6]^ as a combined carboxylate/sulfate cluster as part of a MOF structure in 2015,^[7]^ and lastly as an isolated carboxylate/sulfate cluster in 2018.^[8]^ The hexameric cluster motif is also found in newly reported doubly^[9]^ and five‐fold fused clusters.^[10]^ Zr‐based MOFs can also be obtained with Zr‐coordinated sulfate introduced as a precursor or postsynthetically, and the materials show similar characteristics as sulfated zirconia catalysts.^[11]^

The aqueous chemistry of Zr/Hf sulfates has been studied in detail, and several crystalline species of oligomeric Zr oxysulfates have been described, with and without additional ligands. Notably, the first large oxysulfate, Zr_18_(OH)_26_O_20_(H_2_O)_23.2_(SO_4_)_12.7_ (Zr_18_), was isolated and characterized in 1987.^[12]^ It has recently been found that pre‐nucleation, or the assembly of building units (BUs) in solution, plays a large role in determining which species are found in the solid phase, and that the compositions of these BUs depend on the Zr:SO_4_ ratio.^[13]^ However, it is not fully understood whether the BUs present in solution during the formation of solid Zr oxysulfate clusters have well‐defined compositions and structures, or if their state is more fluid/amorphous.

The Zr_70_ unit reported herein was discovered by serendipity while screening synthesis conditions for promoted Zr‐based catalysts. A hydrothermal reaction between Zirconium(IV) sulfate and Magnesium(II) nitrate yielded large single crystals of an unknown phase with a large unit cell, as evident by sharp low‐angle reflections in its PXRD pattern (Figure S6). Structural analysis by single crystal X‐ray diffraction (SC‐XRD) revealed a complex structure consisting of large toroidal clusters (see Figure 1) with the formula Zr_70_(SO_4_)_58_(O/OH)_146_⋅*x* (H_2_O)⋅[Mg(H_2_O)_6_]_*y*_ hereafter called Zr_70_‐mP‐Mg (where mP signifies a primitive monoclinic crystal lattice). Each toroidal cluster in the Zr_70_‐mP‐Mg crystal structure was linked to two others by two bridging sulfates each, forming a staircase‐like infinite polymeric chain (shown in Figure 2). The chains stack in parallel (symmetric to each other by 2‐fold rotation), in a herringbone‐like packing mode (Figure 3).

**Figure 1:**
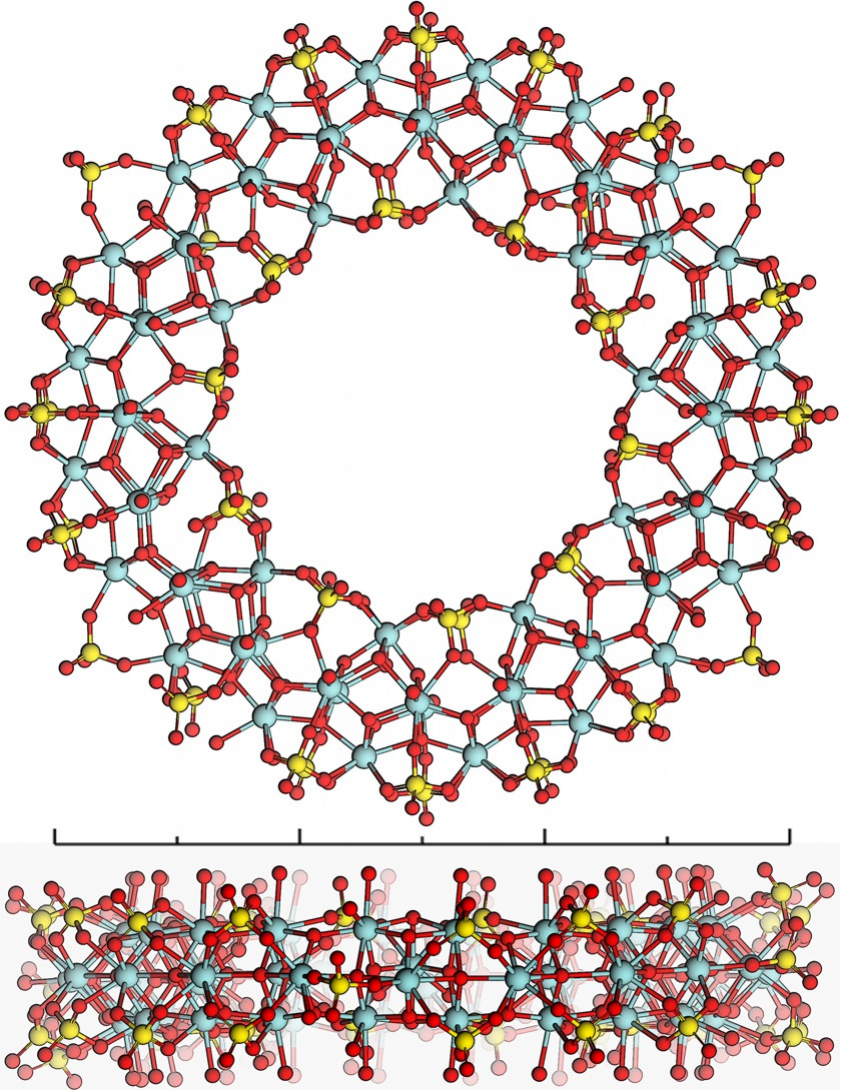
Top: Front view of Zr_70_, showing the overall structure and distribution of different outer rim sulfate arrangements. Bottom: Side view of Zr_70_, showing its thickness. Coloring: Teal: Zr, yellow: S, red: O. Scale bar: 30 Å.

**Figure 2:**
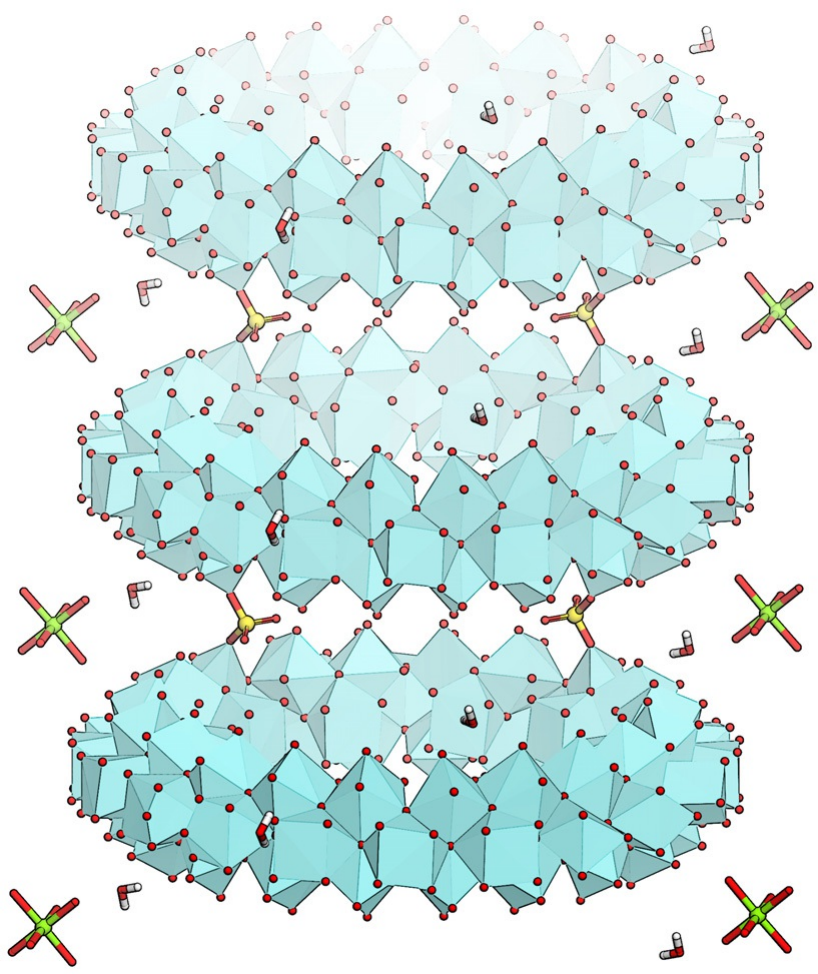
Zr_70_ rings linked by bidentate sulfate forming one‐dimensional chains in Zr_70_‐mP‐Mg. Zr atoms shown as polyhedra. Middle: Teal: Zr, yellow: S, red: O, green: Mg, white: H.

**Figure 3:**
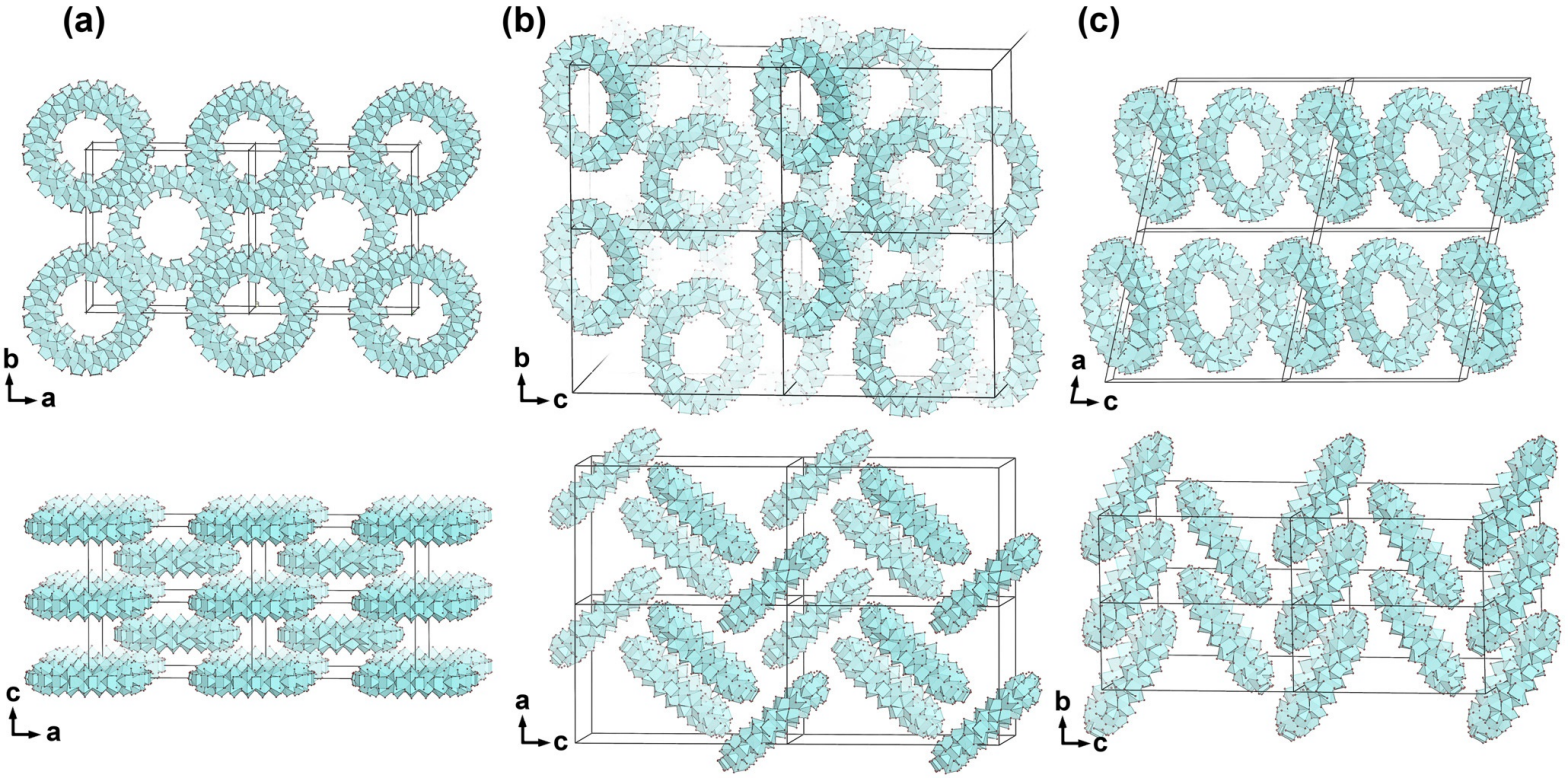
The packing modes of Zr_70_‐rings, a) the tetragonal (tI) packing, where the Zr_70_ rings form layers of parallel rings, with one‐directional circular channels, b) the orthorhombic packing, with nearly perpendicular rings without bridging sulfate ions, and c) the monoclinic/triclinic structure which both consist of one‐dimensional chains of Zr_70_ rings connected by bridging sulfate ions.

Curiously, magnesium is not a part of the ring structure, but occupies the interstitial space as a hexaaqua‐complex stabilized by hydrogen bonds to the neighboring Zr_70_ units. This realization prompted an investigation as to whether the Zr_70_ toroids would form under similar conditions, but with other reagents in addition to Zr^4+^ sulfate. From previous reports it is known that the Zr:SO_4_ ratio should be lower than the 1:2 stoichiometry found in the precursor to promote the formation of oligomeric species in solution. Since the Zr_70_ had formed just by adding magnesium nitrate, it was assumed that other metal nitrates would promote a similar reaction. In such a solution of two salts, the complexation between the secondary metal and sulfate in the solution would adjust the Zr:SO_4_ ratio in situ. In parallel, additions of Zirconium(IV) oxynitrate (ZrO(NO_3_)_2_) was also investigated to obtain solutions with adjusted Zr:SO_4_ ratios without the presence of secondary cations. All syntheses were conducted at hydrothermal conditions (185 °C) using Teflon‐lined steel autoclaves, and the obtained products are summarized in Table 1.

**Table 1:** Overview of the synthesis screening and the obtained products. s: structure determined by SC‐XRD, m: unit cell determined from SC‐XRD, n: nanocrystalline phase also obtained (in separate syntheses).

Co‐crystallizing agent	Observed product^[a]^
Blank (only Zr(SO_4_)_2(aq)_)	No product
ZrO(NO_3_)_2_	Zr_70_‐tI^s,n^
BTPPCl	Zr_70_‐tI^s^
NaNO_3_	Zr_70_‐oP‐Na^s,n^
Al(NO_3_)_3_	Zr_70_‐oP‐Al^s,n^
Mg(NO_3_)_2_	Zr_70_‐mP‐Mg^s,n^
Mn(NO_3_)_2_	Zr_70_‐aP‐Mn^s^
Ni(NO_3_)_2_	Zr_70_‐mP‐Ni^m^
Cu(NO_3_)_2_	Zr_70_‐mP‐Cu^s,n^
Zn(NO_3_)_2_	Zr_70_‐aP‐Zn^s,n^ Zr_70_‐mP‐Zn^s^
LiNO_3_, Ga(NO_3_)_3_, Fe(NO_3_)_3_, In(NO_3_)_3_	Unknown nanocrystalline solid

[a] The abbreviated names contain the bravais lattice of the obtained product: aP: triclinic, mP: primitive monoclinic, oP: primitive orthorhombic, tI: body‐centered tetragonal.

From SC‐XRD structure determination, four phases were identified, consisting of the same toroidal Zr_70_ cluster arranged in different packing modes. The packing is clearly influenced by the identity of the co‐reagent. All M^II^ nitrates direct to a monoclinic (mP) or closely related triclinic (aP) phase in which the Zr_70_ rings are fused to each neighboring unit with two bridging sulfate anions, and the resulting chains packed in a herringbone pattern. Nitrates of sodium and aluminum direct to an orthorhombic (oP) structure in which each torus is close to perpendicular to its nearest neighbor, but without directly bridging units. In all of these cases, metal ions (as aqua‐complexes) and water molecules occupy the interstitial space between the Zr_70_ units. When Zr(SO_4_)_2_ is reacted with ZrO(NO_3_)_2_ or with benzyltriphenylphosphonium chloride (BTPPC, known to co‐crystallize with organic cations), a tetragonal phase (tI) is obtained, in which the rings are arranged in parallel square‐grid layers (Figures 3 and 4).

**Figure 4:**
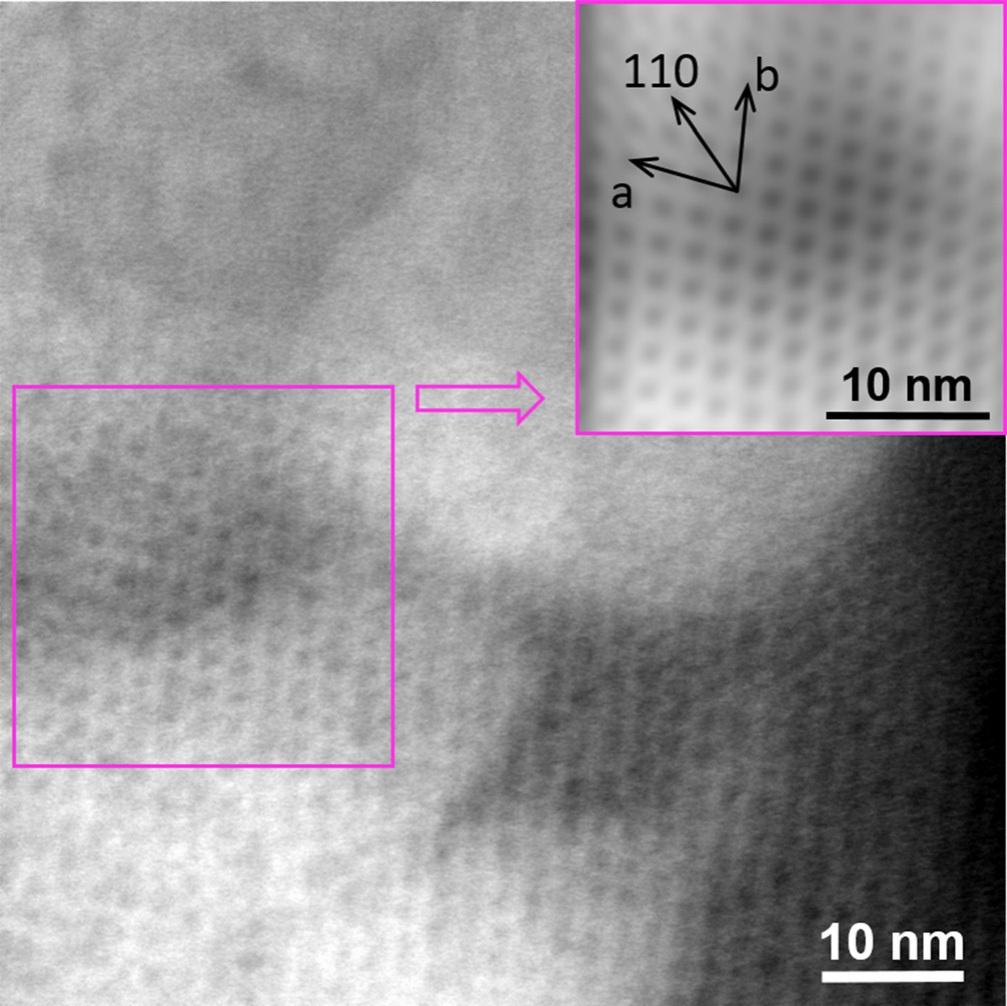
ADF‐STEM image and the corresponding FFT filtered image (inset) of the Zr_70_‐tI structure. A thin layer of the Zr_70_ tetragonal phase was detected, oriented very closely along the [001] viewing direction, showing the arrangement of the stacked rings.

A striking difference between the three phases is the density of the packing of rings, the relative magnitude of which can be derived from the unit cell volumes (see Table 2). The highest density phase, the stacked chains of fused rings, is obtained when M^2+^ cations are present, and is the only phase in which the rings are connected by strong bonds via bridging sulfate ions. The lowest density is found in the Zr_70_‐tI phase, where the only cation present is Zr^4+^. The low density of this phase indicate that the presence of other ions facilitates the denser packing modes, perhaps by balancing the negative surface charge of the Zr_70_ ring.

**Table 2:** The relative density of the Zr_70_ structures, operationalized as the number of tori per unit cell volume and normalized to the highest‐density phase (Zr_70_‐aP‐Zn).

Phase	Relative density
Zr_70_‐tI	0.760
Zr_70_‐oP‐Na	0.834
Zr_70_‐oP‐Al	0.805
Zr_70_‐mP‐Mg	0.914
Zr_70_‐mP‐Cu	0.869
Zr_70_‐aP‐Mn	0.993
Zr_70_‐aP‐Zn	1
Zr_70_‐mP‐Zn	0.993

The structure of the Zr_70_ torus is seemingly identical in all the reported structures, consisting of 10 repeating sub‐units of Zr_7_(SO_4_)_5‐6_O_14_ (Figure 1) related by a 10‐fold rotation axis. The 10 Zr atoms of the inner rim are connected by two parallel bridging sulfate ions each, whereas the 20 Zr of the outer rim of the ring are connected by either singly or doubly bridging sulfate ions, for a total of five or six sulfate per repeating sub‐unit. Determined by the crystal structure refinements, there are 4 to 5 Zr‐Zr pairs in the outer rim connected by a singly bridging (bidentate) sulfate per toroid, whereas the two parallel sulfate that occupy the rest of the outer rim are tridentate (shown in Figure 5). The reason for this disorder is unclear, but could for example originate from the structure of the BUs present in the solution, provide the appropriate charge balance or minimize the strain in the toroid. All structures also contain monodentate sulfate ions on the side of the torus.

Zirconium is predominantly 8‐coordinated in the structure, but some 7‐coordinated species occur in the case of singly‐bridging sulfate groups. The arrangement of Zr atoms within each sub‐unit can be regarded as an octahedron of six Zr‐atoms, with μ_3_‐O or μ_4_‐O capping each facet. To account for the curvature of the toroid, the outer rim of the ring contains an additional Zr bridging the Zr_6_ octahedra (Figures 1 and S1).

In the aP and mP phases, each individual torus is bound to two neighbouring units with two bridging sulfate anions. The single crystals of these phases break into fiber‐like fragments when stressed (e.g. when prodded with a needle), indicating significant anisotropy in its mechanical properties caused by the unidirectional strong bonds. These related phases are the only ones that have not been observed to crystallize at ambient temperature, which suggests that the bridged chains might need hydrothermal conditions to form. By contrast, both Zr_70_‐tI and Zr_70_‐oP‐Na were observed to crystallize by slow evaporation of the mother liquor after hydrothermal treatment. Although this demonstrates the rings’ solubility in a mixture of sulfuric and nitric acid, all Zr_70_ materials reported herein shows very poor solubility in water and ethanol.

From the diffraction data of the Zr_70_‐oP‐Na phase, it was possible to resolve positions of many interstitial atoms (aqua‐sodium complexes and water), showing a network of sodium ions and water between two rings (Figure 5). Only in small parts of the structure is the disorder too large to be resolved. Judging from the positions of the Na^+^ ions, wedged between the anion‐rich surfaces of the rings, they seem to facilitate this packing mode.

**Figure 5:**
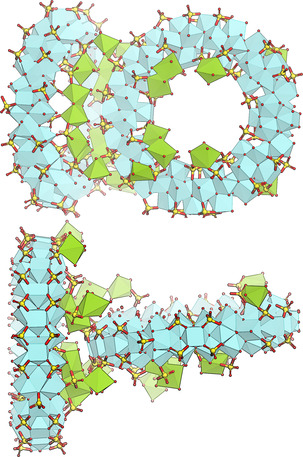
Partial unit cell of Zr_70_‐oP‐Na, from two sides, showing structurally resolved sodium ions (green). The sodium ions whose positions can be determined by diffraction, occupy the regions of close proximity between Zr_70_ rings, presumably facilitating denser packing than in Zr_70_‐tI.

Zr_70_‐tI consists of layers of parallel tori arranged in a square‐grid pattern, co‐planar with the *a*–*b* plane of the unit cell (see Figures 3 and 4). The square‐grid arrangement indicates a structure‐directing interaction between the rings, since other packing modes (e.g. hexagonally arranged layers) would have provided higher density. This is corroborated by the presence of monodentate sulfate ions only present on the axial surfaces that are not in close proximity to neighboring rings. The layers of rings are stacked along the *c*‐axis, alternating in an A‐B‐A‐B configuration where the centroid of each torus of the A layer is aligned with the space between four rings of the B‐layer. This arrangement provides the basis of the tetragonal body‐centered crystal structure.

The Zr_70_‐tI structure shows complex disorder. The diffraction pattern indicates the space group *I*4/*mmm*, but this is not compatible with the point symmetry of Zr_70_: the 4‐fold rotation axis of the space group is co‐aligned with the 10‐fold rotation axis of the torus, and these rotation axes are incompatible. The resulting crystal structure features disordered Zr_70_ units, with (at least) two arrangements of the torus occupying the same volume of the unit cell. To verify that this disorder was intrinsic to the material (and not an artefact related to twinning); more than ten different crystals from three different batches were tested. PXRD refinements also show perfect agreement with *I*4/*mmm* (Figure S5). The symmetry of the Zr_70_ and its crystal structures are further elaborated in the supporting information (see for example, Figure S1).

As previously mentioned, the formation of the rings is likely enabled by a displacement of the Zr:SO_4_ ratio. When a second cation is present in the solution, this could form aqueous complexes with sulfate thus lowering the concentration of available sulfate. When the Zr oxynitrate and sulfate are mixed, the ratio is shifted by the increased concentration of Zr. In the case of Zr sulfate and BTPPCl, a second solid phase of (BTPP)_2_(SO_4_) is formed alongside Zr_70_‐tI, highlighting how sulfate is abstracted by the secondary cation.

One systematic study of species in aqueous solutions across varying Zr:SO_4_ ratios reports multimeric species in solution whose structure depend on this ratio, and finds similar characteristics between the large multimeric species in solution (probed by SAXS and HEXS) and a 18‐meric cluster precipitating from the examined solution.^[13]^ This Zr_18_ cluster has the formula [Zr_18_(OH)_26_O_20_(H_2_O)_23.2_(SO_4_)_12.7_]Cl_0.6_⋅*n* H_2_O, which gives a Zr:SO_4_ ratio of 1:0.7, close to the corresponding ratio of 1:0.8 for Zr_70_. The 18‐mer shows strong structural resemblance of Zr_70_, such as the arrangement of the central Zr atoms and the singly‐ and doubly‐bridging sulfate ions in the outer rim (Figure 6). Although the solution chemistry of Zirconium oxysulfates is well understood at ambient conditions, the hydrothermal conditions seem to enable further oxolation of the oligomeric solution species into rings. These similarities imply that Zr_18_ could be a precursor of Zr_70_, or that the two have common solution precursors.

**Figure 6:**
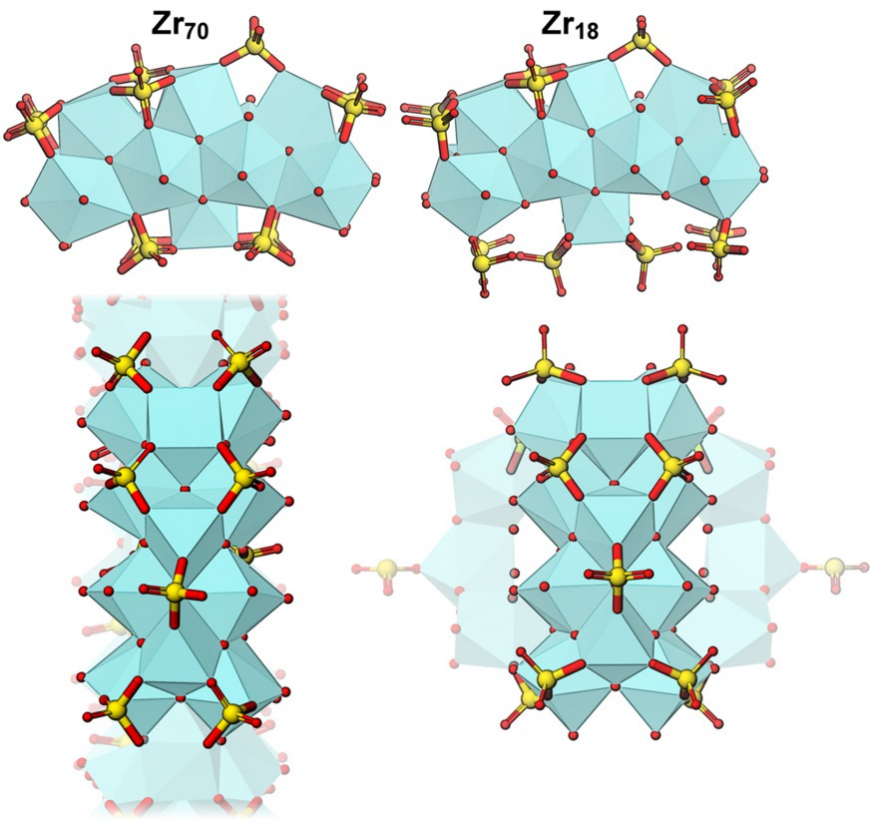
Sub‐units of Zr_70_ (left) and Zr_18_ (right), highlighting the similar structural motif. Of particular interest is the presence of single and double bridging sulfate groups in both complexes. The six peripheral Zr atoms of Zr_18_ are hidden for the sake of clarity (shown transparent in the bottom panel).

The Zr_70_‐tI phase displays infinite cylindrical channels along the c‐axis, of approximately 1 nm in diameter. N_2_ adsorption measurements show a considerable permanent porosity (SA_BET_ of 241 m^2^ g^−1^) which corresponds to a volumetric porosity of around 20 % (based on the solvent accessible pores of an N_2_‐sized probe molecule of the crystal structure). Depending on the size of the adsorbate, the pores are accessible through the holes of the tori, or through smaller pore windows on the sides of the structure (Figure 7). The kinetic diameter of the side windows vary greatly, depending on the conformation of the closest sulfate groups.

**Figure 7:**
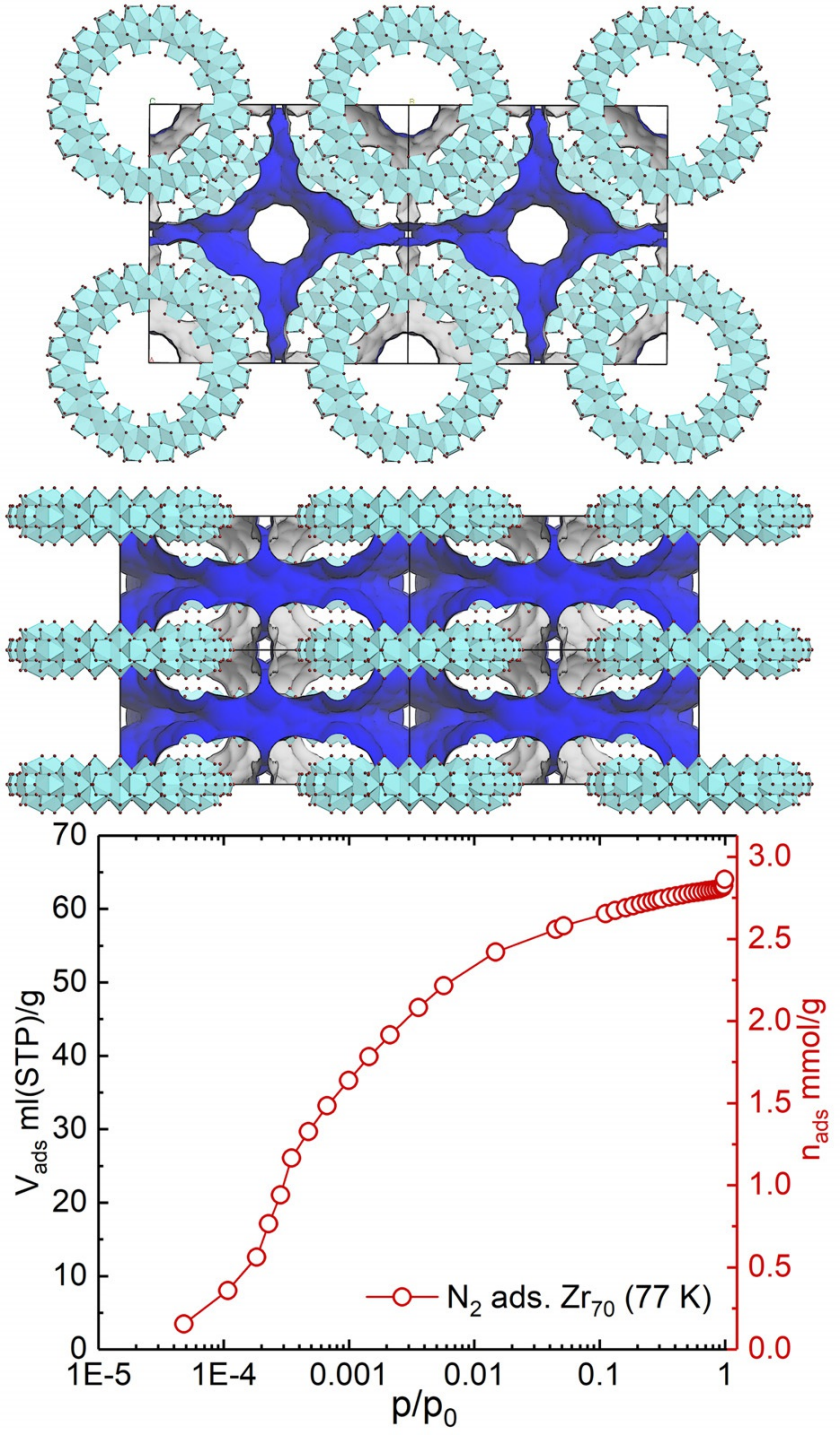
Top: Solvent‐accessible surfaces (blue) in Zr_70_‐tI, showing the surface accessible by a N_2_ molecule. The longitudinal channels are clearly open, but very small molecules might access the pores from the *a* and *b* sides. A large octahedral cavity exists between six neighboring rings. Bottom: N_2_ adsorption isotherm (at 77 K), showing significant microporosity as evident by the sharp uptake at very low partial pressure.

These findings highlight an important difference between Zr_70_ and the recently reported isostructural U_70_, since different BUs have been identified in the two systems. In the case of Uranium, the anionic species [U_6_O_4_(OH)_4_(SO_4_)_12_]^12−^ can be isolated. Although numerous hexameric Zr units are known, the dodecasulfate has not yet been reported.

An important requirement for widespread application of a material is the ability to control its phase and morphology, by identifying the factors that govern it. One such example is provided with “POMzites”, which consists of ring‐shaped POMs (P_8_W_48_O_184_)^40−^ linked by transition metal ions which direct different network structures.^[14]^ It is likely that further ring packing topologies of Zr_70_ will be discovered in the future, analogous to how 14 different ring packing modes have been discovered for the POMzites.

In summary, we have reported a new, inorganic toroidal Zr_70_ oxysulfate cluster that can form a variety of phases, one of which exhibits significant permanent porosity. Albeit structurally complex, Zr_70_ consists of simple building units and is very simple to synthesize. This Zr_70_ oxysulfate cluster likely forms by oxolation of already known oligomeric species, following in situ adjustment of the Zr:SO_4_ ratio in the solution. The diversity of packing structures exhibited by the Zr_70_ units, including one with considerable permanent microporosity, in combination with the described structure‐directing parameters (i.e. the nature of the co‐reacting metal ion and hydrothermal conditions) may provide a robust platform for the development of new, Zr_70_‐based materials with novel functional properties.

## Conflict of interest

A patent application concerning the reported structures has been filed by the University of Oslo with inventors S.Ø. and U.O.

## Supporting information

As a service to our authors and readers, this journal provides supporting information supplied by the authors. Such materials are peer reviewed and may be re‐organized for online delivery, but are not copy‐edited or typeset. Technical support issues arising from supporting information (other than missing files) should be addressed to the authors.

Supplementary
