# Supplementary material for: A Toroidal Zr70 Oxysulfate Cluster and Its Diverse Packing Structures
Source: Angew Chem Int Ed Engl. 2020 Nov 11;59(48):21397–402. doi: 10.1002/anie.202010847 (PMC7756470; doi:10.1002/anie.202010847)
Supplement: Supplementary file 1 — Supplementary [file ANIE-59-21397-s001.pdf]

Angewandte Chemie
Eine Zeitschrift der Gesellschaft Deutscher Chemiker

Supporting Information

A Toroidal Zr_{70} Oxysulfate Cluster and Its Diverse Packing Structures

Sigurd Øien-Ødegaard, Calliope Bazioti, Evgeniy A. Redekop, Øystein Prytz, Karl Petter Lillerud, and Unni Olsbye*

anie_202010847_sm_miscellaneous_information.pdf

Supporting Information

Table of Contents

Experimental Procedures 1

Results and Discussion 5

References 14

Author Contributions 14

Experimental Procedures

All chemicals were obtained in reagent grade from the following sources and used without further purification: SigmaAldrich: $ZrO(NO_3)_2 \cdot xH_2O$, benzyltriphenylphosphonium chloride (BTPPCl), $Al(NO_3)_3 \cdot 9H_2O$, $Mg(NO_3)_2 \cdot 6H_2O$, $Mn(NO_3)_2 \cdot 4H_2O$, $Ni(NO_3)_2 \cdot 6H_2O$, $Cu(NO_3)_2 \cdot 3H_2O$, $Zn(NO_3)_2 \cdot 6H_2O$, $La(NO_3)_3$, $Gd(NO_3)_3$ and $(NH_4)_2Ce(NO_3)_6$. VWR: $NaNO_3$. AlfaAesar: $Zr(SO_4)_2 \cdot 4H_2O$.

The synthesis screening study following the initial discovery of Zr_{70}-mP-Mg was conducted by reacting a series of metal nitrates with Zr(IV)sulfate (see below for detailed procedures for all samples), under hydrothermal conditions in Teflon-lined steel autoclaves at 185 °C. The resulting solid (if any) was analyzed with powder and single crystal (if applicable) X-ray diffraction and thermogravimetric analysis (TGA). Several of the products were also subject to SEM/EDX and N_2 adsorption analysis. In the case of Zr_{70}-oP-Na and Zr_{70}-tI, the single crystals were grown at room temperature postsynthetically by slow evaporation, whereas the other investigated single crystals and powders were obtained from the hydrothermal synthesis directly.

For most of the screened co-reagents, single crystals suitable for analysis could be obtained by adjusting the stoichiometric ratio between the reagents, usually around a ratio between 2:1 and 1:2 of [Zr]:[$M(NO_3)_x$]. When even higher concentrations of Zr ([Zr]>[$M(NO_3)_x$]) were used, an unidentified phase consisting of sub-micrometer thin plates was obtained in most cases (samples marked with n in Table 1). The two phases were never obtained in the same synthesis, except in the case of Zr_{70}-oP-Na, where the nanocrystalline phase was obtained in the hydrothermal synthesis, and octahedral single crystals were obtained by slow evaporation of the supernatant post synthesis.

Single crystals were mounted directly from the mother liquor and transferred as quickly as possible to a cryogenic nitrogen gas stream in the diffractometer before measurement. Upon prolonged exposure to ambient air, peak broadening and loss of resolution was observed in the X-ray diffraction patterns. This is assumed to be related to the evaporation of water molecules occupying the interstitial space between the toroidal Zr_{70} units, analogous to crystal water in protein crystals. Inhomogeneous distribution of water throughout the single crystal (i.e. when near-surface water molecules evaporate at a higher rate) breaks the long-range order of the crystal and thus loss of diffraction signal. During structure refinement of the SC-XRD data, the interstitial species, i.e. metal aqua complexes and crystal water present in all samples, were added to the model until the displacement parameters became unphysically large and/or the refinement unstable. When this limit was reached, a solvent mask (PLATON/SQUEEZE) was applied to compute the contribution of disordered metal ions and water molecules to the calculated structure factors.[1] CIF files containing all structural information can be downloaded from the Inorganic Crystal Structure Database (ICSD), submission numbers CSD 2003566-2003573.

SC-XRD

SC-XRD data sets were acquired using a Bruker D8 Venture diffractometer with Mo and Cu Kα radiation. Diffraction experiments were performed on samples extracted directly from the mother liquor, and the sample was kept in a stream of dry nitrogen at 100 K during measurement. Unit cell determination, data collection, integration and scaling were performed using the Bruker APEX3 suite (version 2019.1-0).[2] The structures were solved using XT,[3] with the exception

of Zr_{70}-aP-Zn that was solved using XS.[4] The structures were refined using XL[5] with OLEX2 as GUI.[6] Figures were made with Biovia MaterialsStudio 2019.

N_2 Adsorption

The nitrogen sorption isotherm was acquired using a BelSorp mini II instrument. Around 100 mg of Zr_{70}-tI powder sample was weighed into a 9 cm^3 glass cell, pretreated at 80 °C for 30 min and 300 °C for 90 minutes under vacuum, before the isotherm was recorded at 77 K in the pressure range of 0 - 1.0 p/p_0 (where p_0 is 1 atm). The BET surface area of 241 m^2/g and the corresponding micropore volume of 55 cm^3/g were calculated using established consistency criteria for microporous materials,[7] from the pressure range p/p_0: 0.00005 to 0.04478.

TGA

TGA was performed using a Netzsch Jupiter F3 STA. The ramp rate was set to 5 °C/min, measuring weight loss and heat (DSC) in the range of 25 – 900 °C, in an atmosphere of synthetic air 20 $\%_{vol}$ O_2 in N_2.

SEM/EDX

SEM images were taken on a Hitachi SU8230 Field Emission Scanning Electron Microscope (FE-SEM). A small amount of the measured samples were dried at 110 °C and sprinkled on an aluminium sample holder fitted with carbon adhesive.

TEM

(Scanning) Transmission Electron Microscopy (S)TEM investigations were conducted on a FEI Titan G2 60-300 kV equipped with a CEOS DCOR probe-corrector. Observations were performed at 300 kV with a probe convergence angle of 24 mrad. The camera length was set at 77 mm and simultaneous STEM imaging was conducted with 3 detectors: high-angle annular dark-field (HAADF) (collection angles 98.7-200 mrad), annular dark-field (ADF) (collection angles 21.5-98.7 mrad) and annular bright-field (ABF) (collection angles 10.6 - 21.5 mrad). The resulting spatial resolution achieved was approximately 0.08 nm. Fast Fourier Transform (FFT) analysis and filtering was performed in high-resolution STEM images. To avoid possible beam damage, imaging of very thin-promising areas was performed immediately, without precise titling of the sample along specific crystallographic directions.

The Zr_{70}-tI sample was prepared by dispersing a small amount of the dried sample in ethanol, and agitate this suspension in an ultrasound bath for 30 minutes. A droplet of the suspension was applied to a copper grid, and the ethanol was allowed to evaporate.

PXRD

Samples were prepared using ~30 mg of the sample (wet from mother liquor) on a glass plate fitted sample holder by spreading the crystalline powder in a uniform layer atop the glass plate, and covering with transparent plastic film. The plastic film gives a small signal in the PXRD patterns observed at 2θ =~22° and 34° as broad peaks, which have been refined as a separate phase. All patterns were acquired using Cu Kα radiation, λ = 1.5418. Refinements for Zr_{70}-tI were performed using the structure determined by SC-XRD. In the Rietveld refinement, atom positions were kept static, but other parameters were allowed to refine.

Synthesis

Zr_{70}-tI microcrystalline

To 20 mL distilled water, 4.0 g $Zr(SO_4)_2$•$4H_2O$ was dissolved under stirring at room temperature (11 mmol Zr, 23 mmol SO_4^{2-}). To this solution 5.0 g $ZrO(NO_3)_2$•xH_2O was added (14-16 mmol Zr*), and a clear colorless solution was obtained. This was transferred to a teflon-lined steel autoclave and heated to 185 °C, at which it was kept for 24 hours, before slowly cooled to RT. A white crystalline product was recovered by filtration.

*: The $ZrO(NO_3)_2•xH_2O$ has a Zr content of 25 – 30 % by weight (from the certificate of analysis provided by the manufacturer) causing the uncertainty in Zr content.

Zr_{70}-tI single crystals

To 20 mL distilled water, 4.0 g $Zr(SO_4)_2•4H_2O$ was dissolved under stirring at room temperature (11 mmol Zr, 23 mmol SO_4^{2-}). To this solution 2.0 g benzyltriphenylphosphonium chloride (BTPPCl) was added, and a clear colorless solution was obtained. This was transferred to a teflon-lined steel autoclave and heated to 185 °C, at which it was kept for 24 hours, before slowly cooled to RT. Large colorless crystals were observed and kept in the mother liquor after synthesis. Precipitation of more, large colorless crystals continued over the following 2-5 days.

By visual inspection in microscope, two distinct phases could be observed. The majority of the initial precipitate was $[BTPP]_2SO_4$. Single crystals of Zr_{70}-tI continued to precipitate from the mother liquor, and crystal growth could be monitored visually in an optical microscope.

Zr_{70}-oP-Na powder

To 20 mL distilled water, 4.0 g $Zr(SO_4)_2•4H_2O$ was dissolved under stirring at room temperature (11 mmol Zr, 23 mmol SO_4^{2-}). To this solution 2.87 g $NaNO_3$ was added (33.8 mmol), and a clear colorless solution was obtained. This was transferred to a teflon-lined steel autoclave and heated to 185 °C, at which it was kept for 24 hours, before slowly cooled to RT. A white crystalline product was recovered by filtration.

Zr_{70}-oP-Na nanocrystals and single crystals

To 20 mL distilled water, 4.0 g $Zr(SO_4)_2•4H_2O$ was dissolved under stirring at room temperature (11 mmol Zr, 23 mmol SO_4^{2-}). To this solution 1.44 g $NaNO_3$ was added (16.9 mmol), and a clear colorless solution was obtained. This was transferred to a teflon-lined steel autoclave and heated to 185 °C, at which it was kept for 24 hours, before slowly cooled to RT. A white nanocrystalline product was recovered by filtration. Upon storage of the mother liquor at ambient conditions, large octahedral crystals precipitated over the next 5-10 days.

Zr_{70}-oP-Al

The synthesis was performed in an array autoclave with 24 teflon-lined wells of 2 mL. To 2 mL distilled water, 0.40 g $Zr(SO_4)_2•4H_2O$ was dissolved under stirring at room temperature (1.1 mmol Zr, 2.3 mmol SO_4^{2-}). To this solution, 0.20 g $Al(NO_3)_3•9H_2O$ was added (0.53 mmol), and a clear colorless solution was obtained. This was transferred to a teflon-lined steel autoclave-well and heated to 185 °C, at which it was kept for 24 hours, before slowly cooled to RT. A white crystalline product was recovered by filtration. This procedure yielded the crystal from which the structure was determined, but has successfully been scaled up to 20 mL.

Zr_{70}-mP-Mg

The synthesis was performed in an array autoclave with 24 teflon-lined wells of 2 mL. To 2 mL distilled water, 0.40 g $Zr(SO_4)_2•4H_2O$ was dissolved under stirring at room temperature (1.1 mmol Zr, 2.3 mmol SO_4^{2-}). To this solution, 0.20 g $Mg(NO_3)_2•6H_2O$ was added (0.78 mmol), and a clear colorless solution was obtained. This was transferred to a teflon-lined steel autoclave-well and heated to 185 °C, at which it was kept for 24 hours, before slowly cooled to RT. A white crystalline product was recovered by filtration. This procedure yielded the crystal from which the structure was determined, but has successfully been scaled up to 20 mL.

Zr_{70}-aP-Mn

To 20 mL distilled water, 4.0 g $Zr(SO_4)_2•4H_2O$ was dissolved under stirring at room temperature (11 mmol Zr, 23 mmol SO_4^{2-}). To this solution 5.5 g $Mn(NO_3)_2•4H_2O$ was added (22 mmol), and a clear, pale pink solution was obtained. This was transferred to a teflon-lined steel autoclave and heated to 185 °C, at which it was kept for 24 hours, before slowly cooled to RT. A pale pink crystalline product was recovered by filtration. The yield was remarkably low,

Zr_{70}-mP-Ni

To 20 mL distilled water, 4.0 g $Zr(SO_4)_2$•$4H_2O$ was dissolved under stirring at room temperature (11 mmol Zr, 23 mmol SO_4^{2-}). To this solution 4.9 g $Ni(NO_3)_2$•$6H_2O$ was added (17 mmol), and a clear, pale green solution was obtained. This was transferred to a teflon-lined steel autoclave and heated to 185 °C, at which it was kept for 24 hours, before slowly cooled to RT. A pale green crystalline product was recovered by filtration.

Zr_{70}-mP-Cu

To 20 mL distilled water, 4.0 g $Zr(SO_4)_2$•$4H_2O$ was dissolved under stirring at room temperature (11 mmol Zr, 23 mmol SO_4^{2-}). To this solution 4.0 g $Cu(NO_3)_2$•$3H_2O$ was added (17 mmol), and a clear, blue solution was obtained. This was transferred to a teflon-lined steel autoclave and heated to 185 °C, at which it was kept for 24 hours, before slowly cooled to RT. A pale blue crystalline product was recovered by filtration.

Zr_{70}-aP-Zn

To 20 mL distilled water, 4.0 g $Zr(SO_4)_2$•$4H_2O$ was dissolved under stirring at room temperature (11 mmol Zr, 23 mmol SO_4^{2-}). To this solution 2.0 g $Zn(NO_3)_2$•$6H_2O$ was added (6.7 mmol), and a clear, colorless solution was obtained. This was transferred to a teflon-lined steel autoclave and heated to 185 °C, at which it was kept for 24 hours, before slowly cooled to RT. A white crystalline product was recovered by filtration.

Zr_{70}-mP-Zn

To 20 mL distilled water, 5.0 g $Zr(SO_4)_2$•$4H_2O$ was dissolved under stirring at room temperature (14 mmol Zr, 28 mmol SO_4^{2-}). To this solution 5.0 g $Zn(NO_3)_2$•$6H_2O$ was added (17 mmol), and a clear, colorless solution was obtained. This was transferred to a teflon-lined steel autoclave and heated to 185 °C, at which it was kept for 24 hours, before slowly cooled to RT. A white crystalline product was recovered by filtration.

Other attempts

$La(NO_3)_3$, $Gd(NO_3)_3$ and $(NH_4)_2Ce(NO_3)_6$ were also part of the screening experiment, and a viscous liquid product was obtained in all cases, which was easily separated from the mother liquor. When this liquid was exposed to air or liquid water, it solidified to an amorphous solid.

Results and Discussion

SC-XRD

All of the Zr_{70} structures are severely disordered, most prominently by alternating singly and doubly bridging sulfate groups on the outer rim of the toroidal unit. Throughout the structure refinements, the sum of the site occupancy coefficients of these disordered groups has been set to 1 (i.e. every Zr - Zr bridging section of the torus contains either one central or two peripheral bridging sulfate groups). The ratio between the groups were in all cases allowed to refine freely.

In the special case of Zr_{70}-tI, the most likely point symmetry of the Zr_{70} torus (D_{10h}) is not compatible with the point symmetry of the site (D_{4h}) it occupies in the crystal structure (the centroid of the ring is located on the lattice nodes in space group I4/mmm).

Considering only the Zr atoms, these can be divided into three groups according to their radial distance to the centroid of the ring: 10 inner atoms, 40 central atoms and 20 outer atoms. Of these, the central and outer atoms display a (pseudo)-20-fold rotation symmetry, whereas the inner 10 atoms have a (pseudo)- 10-fold symmetry. In Zr_{70}-tI, the 10 inner atoms are disordered over 20 sites at the same angles as the outer 20 atoms. The disorder could be explained by a number of rotations, for example a two-fold rotation around the a or b axes, or any rotation around the c axis changing the positions of the inner Zr atoms (figure S1). Attempts to solve the structure in lower symmetry space groups (e.g. I2/m) did not resolve the ambiguity of conformation.

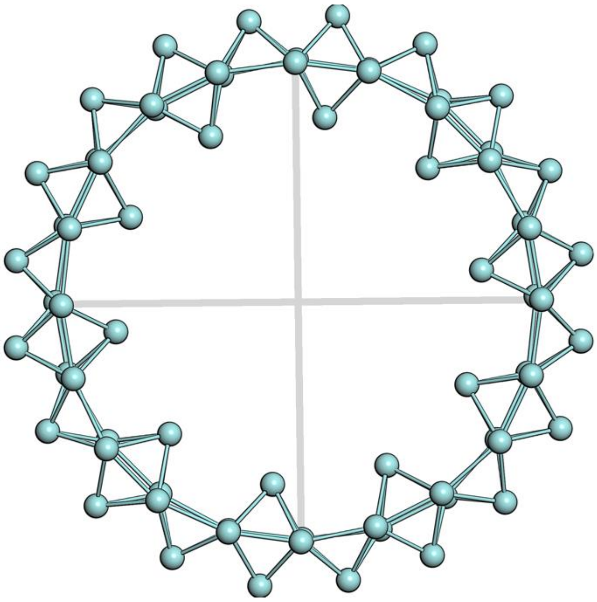

Figure S1. Left: The structure of Zr_{70}, showing only the Zr atoms. Right: Superimposed structures (in yellow and purple) of Zr_{70} (only Zr shown), displaying the positional disorder of the inner Zr atoms. The black diamond signifies the four-fold rotation axis present through the center of the ring in the crystal structure, perpendicular to the plane of view. The grey lines are showing the a and b axes of the unit cell.

Zr_{70}-mP-Ni

Unit cell constants of a = 33.593(18) Å, b = 18.337(9) Å, c = 40.08(2) Å, β = 102.99(5)°, volume = 24060.(30) $Å^3$, are based upon the refinement of the XYZ-centroids of 130 reflections above 20 σ(I) with 2.412° < 2θ < 28.52°. The reflections were harvested from two Ω-scans of 6°, separated by 120° φ, using Mo Kα radiation (λ = 0.71073 Å). Due to the low resolution of the screened crystals, at best corresponding to a d-spacing of 1.4 Å, a full data collection was not performed.

Zr_{70}-aP-Zn

From the collected SC-XRD data, the reflections could be manually sorted to two separate lattices using CELL_NOW. Comparing the orientations of the unit cells, the twin law was found to be a two-fold rotation about the a-axis.

Output from unit cell comparison (Bruker APEX3):

New Cell: a=18.5097 b=31.3582 c=39.5711 alpha=78.757 beta=89.754 gamma=86.992

Figure of Merit (0=ideal) : 7.80
Rotation angle (degrees) :-179.974
Rotation vector (laboratory) : -0.7728 0.1133 0.6245
Rotation vector (reciprocal cell) : -1.00 -0.05 -0.00
Rotation vector (direct cell) : -1.00 0.00 0.00

Superposition matrix : H' = +1.005 * H -0.008 * K
K' = +0.120 * H -1.017 * K -0.001 * L
L' = +0.009 * H -0.007 * K -0.998 * L

Zr_{70}-aP-Mn

From the collected SC-XRD data, the reflections could be manually sorted to two separate lattices using CELL_NOW. Comparing the orientations of the unit cells, the twin law was found to be a two-fold rotation about the a-axis.
Output from unit cell comparison (Bruker APEX3):

New Cell: a=18.2547 b=31.3665 c=39.7196 alpha=102.169 beta=90.134 gamma=92.053

Figure of Merit (0=ideal) : 3.20
Rotation angle (degrees) :-180.018
Rotation vector (laboratory) : 0.8089 0.2051 0.5511
Rotation vector (reciprocal cell) : -16.00 1.00 0.00
Rotation vector (direct cell) : -1.00 -0.00 -0.00

Superposition matrix : H' = +0.994 * H +0.003 * K +0.001 * L
K' = -0.121 * H -0.995 * K
L' = -0.002 * H -0.996 * L
==Main Text Paragraph.

Structure of (BTPP)HSO_4

Table S1. Summary of crystallographic data for (BTPP)HSO_4 collected at ambient temperature.

**Crystal data**	**Below: Molecular structure of (BTPP)HSO_4**
**Chemical formula**	$HO_4S{\cdot}C_{25}H_{22}P$
**M_r**	450.46
**Crystal system, space group**	Monoclinic, C2/c
**Temperature (K)**	297
**a, b, c (Å)**	22.7771 (16), 9.9984 (7), 19.9588 (15)
**β (°)**	90.797 (2)
**V (Å³)**	4544.9 (6)

**Z**	8	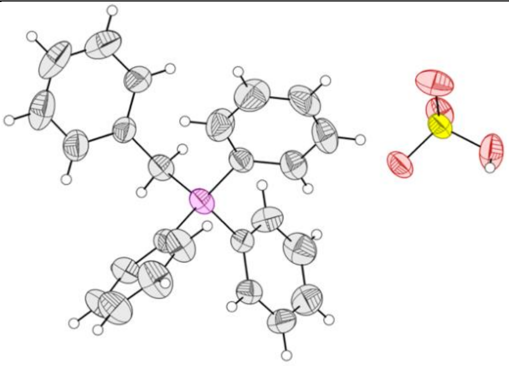
**Radiation type**	Mo Kα	
**μ (mm⁻¹)**	0.24	
**Crystal size (mm)**	0.25 × 0.18 × 0.16	
**Data collection**		
**Diffractometer**	Bruker D8 Venture, CMOS detector	
**Absorption correction**	Multi-scan	
	SADABS2016/2 (Bruker,2016/2) was used for absorption correction. wR2(int) was 0.0664 before and 0.0479 after correction. The Ratio of minimum to maximum transmission is 0.9432. The λ/2 correction factor is Not present.	
**T_{min}, T_{max}**	0.702, 0.745	
**No. of measured, independent and observed [$I > 2\sigma_{(I)}$] reflections**	8810, 2719, 2101	
**R_{int}**	0.036	
**θ_{max} (°)**	22	
**$(\sin \theta/\lambda)_{max}$ (Å^{-1})**	0.527	
**Refinement**		
**$R[F^2 > 2\sigma(F^2)]$, $wR(F^2)$, S**	0.052, 0.116, 1.09	
**No. of reflections**	2719	
**No. of parameters**	281	
**H-atom treatment**	H-atom parameters constrained	
**$\Delta\rho_{max}$, $\Delta\rho_{min}$ (e Å^{-3})**	0.61, −0.35	

Table S2. Summary of crystallographic data for the reported Zr_{70} compounds, collected at 100 K.

**Crystal data**	Zr_{70}-tI	Zr_{70}-oP-Na	Zr_{70}-oP-Al	Zr_{70}-mP-Mg
**CCDC Entry**	2003568	2003573	2003570	2003569
Chemical formula	$O_{385.13}S_{60.42}Zr_{70}$·22.2(O)	$Na_{21.5}O_{450.7}S_{67.3}Zr_{70}$·3.5(O)	$Al_6O_{420.15}S_{69.68}Zr_{70}$·2(AlO2)·19.5(O)	$O_{378.6}S_{58.3}Zr_{70}$·2(MgO6)·4(H2O)
M_r	14855.74	16304.7	15939.1	14624.78
Crystal system, space group	Tetragonal, I4/mmm	Orthorhombic, Pnma	Orthorhombic, Pnma	Monoclinic, $P2_1/c$
Temperature (K)	100	100	100	100
a, b, c (Å)	39.7166 (10), 39.7166 (10), 18.4102 (7)	30.6953 (15), 36.6024 (17), 47.060 (2)	31.2744 (7), 37.1363 (9), 47.1646 (12)	33.5006 (10), 18.4578 (6), 39.5475 (12)
α, β, γ (°)	90, 90, 90	90, 90, 90	90, 90, 90	90, 99.7089 (14), 90
V (Å^3)	29040.4 (18)	52872 (4)	54778 (2)	24103.8 (13)
Z	2	4	4	2
Radiation type	Mo Kα	Mo Kα	Cu Kα	Cu Kα
μ (mm^{-1})	1.50	1.71	14.02	15.22
Crystal size (mm)	0.42 × 0.42 × 0.35	0.26 × 0.25 × 0.21	0.57 × 0.32 × 0.29	0.72 × 0.28 × 0.15
**Data collection**				
Diffractometer	Bruker D8 Venture, CMOS detector			
Absorption correction	Multi-scan			
T_{min}, T_{max}	0.693, 0.746	0.627, 0.745	0.471, 0.749	0.318, 0.751
No. of measured, independent and observed [I > 2σ(I)] reflections	148461, 12495, 10037	230115, 51029, 35585	203315, 24693, 17084	206119, 32348, 28546
R_{int}	0.036	0.047	0.095	0.045
$θ_{max}$ (°)	31.1	25.7	47.3	57
$(\sin θ/λ)_{max}$ (Å^{-1})	0.727	0.611	0.476	0.544
**Refinement**				
R[F^2 > 2σ(F^2)], wR(F^2), S	0.061, 0.212, 1.02	0.074, 0.228, 1.04	0.057, 0.158, 1.05	0.037, 0.086, 1.05
No. of reflections	12495	51029	24693	32348
No. of parameters	533	3019	2883	2438
No. of restraints	125	268	345	54
$Δρ_{max}$, $Δρ_{min}$ (e Å^{-3})	2.27, −2.00	4.01, −1.71	2.36, −0.98	1.95, −0.91

Table S2. (cont.)

**Crystal data**	Zr_{70}-mP-Cu[a]	Zr_{70}-aP-Mn	Zr_{70}-aP-Zn	Zr_{70}-mP-Zn[a]
**CCDC Entry**	2003572	2003571	2003566	2003567
Chemical formula	$O_{375.55}S_{58.55}Zr_{70}\cdot 6(O)$	$O_{373.7}S_{59.05}Zr_{70}\cdot 15(O)\cdot S$	$O_{362.04}S_{56.7}ZnZr_{70}\cdot OZn\cdot 11(O)$	$O_{373.74}S_{56.91}Zr_{70}$
M_r	14367.3	14545.91	14318.5	14189.86
Crystal system, space group	Monoclinic, $P2_1/c$	Triclinic, P-1	Triclinic, P-1	Monoclinic, $P2_1/c$
Temperature (K)	100	100	100	100
a, b, c (Å)	34.860 (3), 18.5387 (16), 40.213 (4)	18.267 (4), 31.355 (6), 39.662 (8)	18.367 (4), 30.989 (6), 39.498 (8)	31.267 (2), 18.3953 (15), 39.411 (4)
α, β, γ (°)	90, 102.337 (2), 90	101.944 (4), 90.023 (5), 92.047 (5)	79.003 (5), 89.872 (4), 88.246 (5)	90, 101.570 (2), 90
V ($Å^3$)	25388 (4)	22210 (7)	22058 (7)	22207 (3)
Z	2	2	2	2
Radiation type	Mo Kα	Mo Kα	Mo Kα	Mo Kα
μ (mm^{-1})	1.71	1.96	2.06	1.94
Crystal size (mm)	1.13 × 0.45 × 0.29	0.68 × 0.21 × 0.16	0.73 × 0.28 × 0.19	0.85 × 0.36 × 0.17
**Data collection**				
Diffractometer	Bruker D8 Venture, CMOS detector			
Absorption correction	Multi-scan			
T_{min}, T_{max}	0.559, 0.745	0.464, 0.745	0.422, 0.745	0.481, 0.745
No. of measured, independent and observed [I > 2σ(I)] reflections	319953, 52370, 38408	159080, 64000, 41299	95340, 54690, 39387	67923, 29352, 21484
R_{int}	0.073	0.103	0.041	0.05
$θ_{max}$ (°)	26.5	23.8	22.8	22.8
$(\sin θ/λ)_{max}$ ($Å^{-1}$)	0.628	0.568	0.545	0.544
**Refinement**				
$R[F^2 > 2σ(F^2)]$, $wR(F^2)$, S	0.087, 0.201, 1.04	0.120, 0.342, 1.03	0.202, 0.557, 2.39	0.212, 0.490, 1.03
No. of reflections	52370	64000	54690	29352
No. of parameters	2523	2926	2662	1233
No. of restraints	174	54	468	0
$Δρ_{max}$, $Δρ_{min}$ (e $Å^{-3}$)	2.97, −1.70	1.99, −1.56	7.33, −2.72	2.74, −2.61

[a]: The counter-ion could not be unambiguously refined in the crystal structure, and is assumed to be disordered in the disordered areas (masked by PLATON/SQUEEZE).

TGA

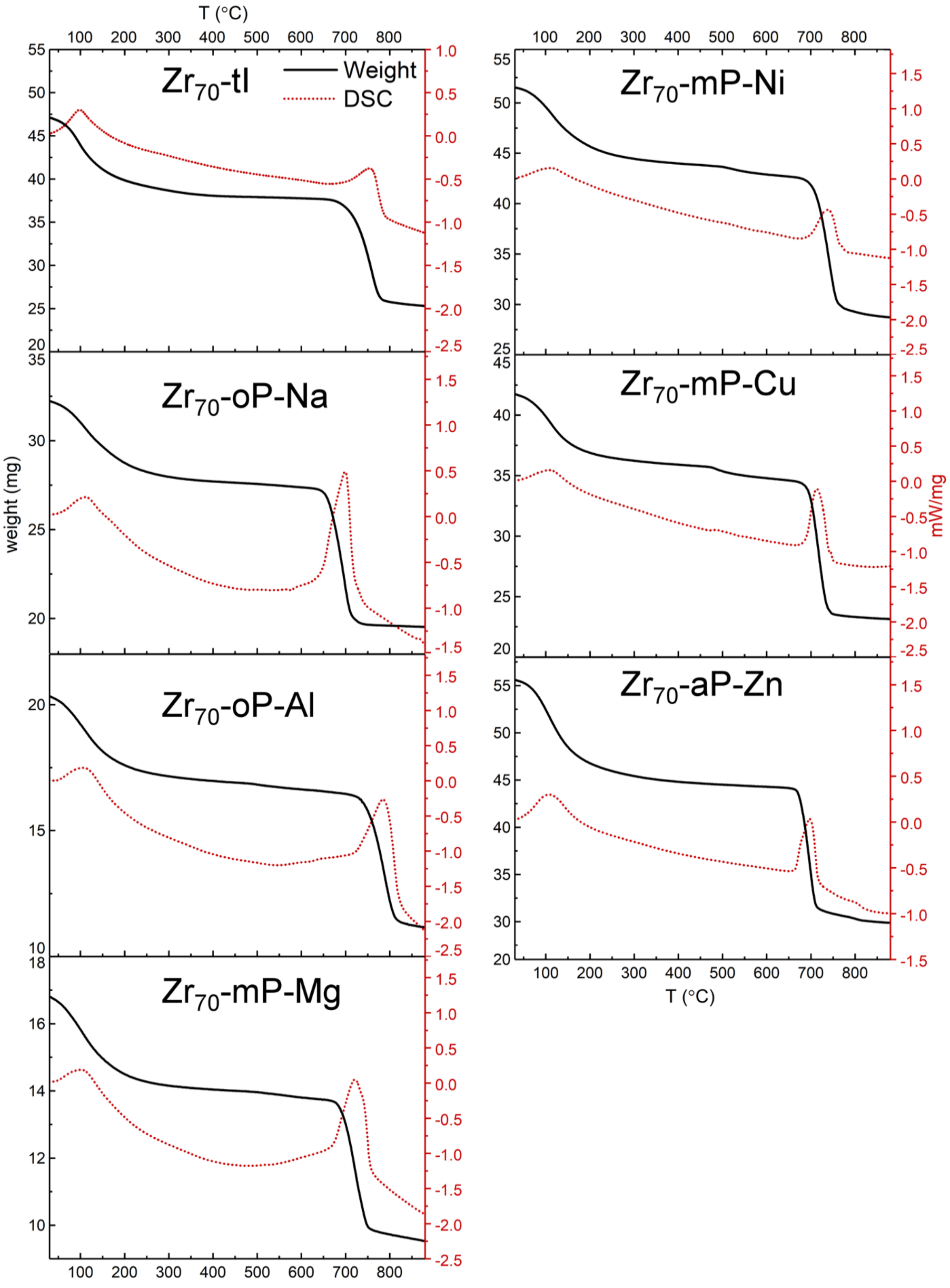

Figure S2. TGA and DSC plots for the reported Zr_{70} phases. All of the phases show two main weight losses accompanied by an endothermic signal. The first signal is associated with the loss of physisorbed water, and the second signal is associated with the loss of sulfate groups as gaseous decomposition products (SO_x).

SEM

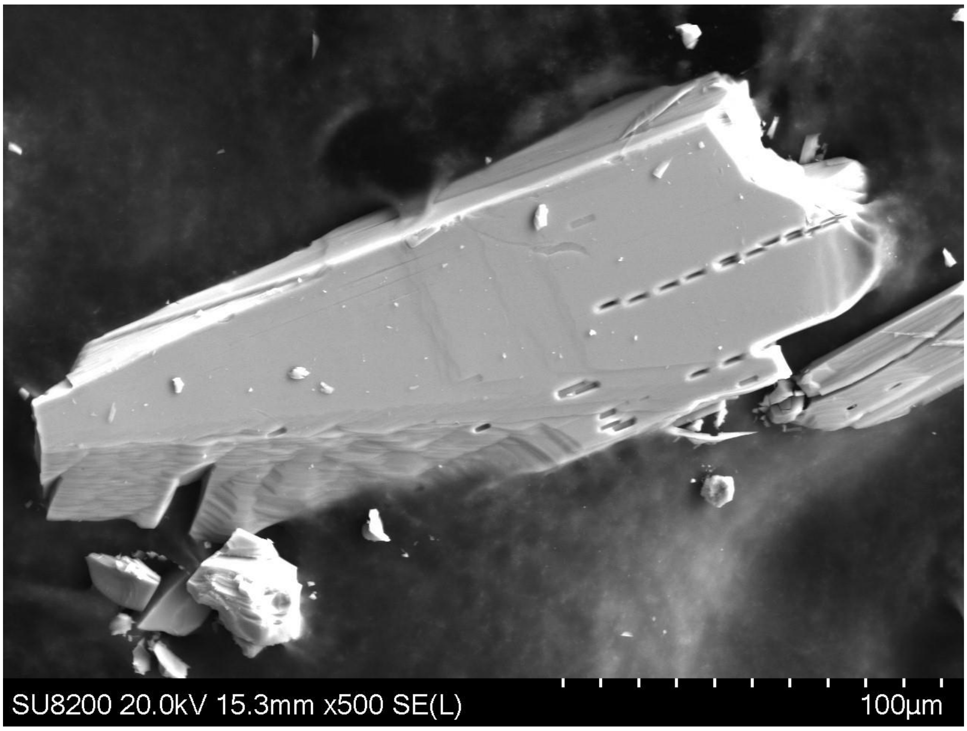

Figure S3. SEM image of Zr_{70}-mP-Cu, showing a large single crystal.

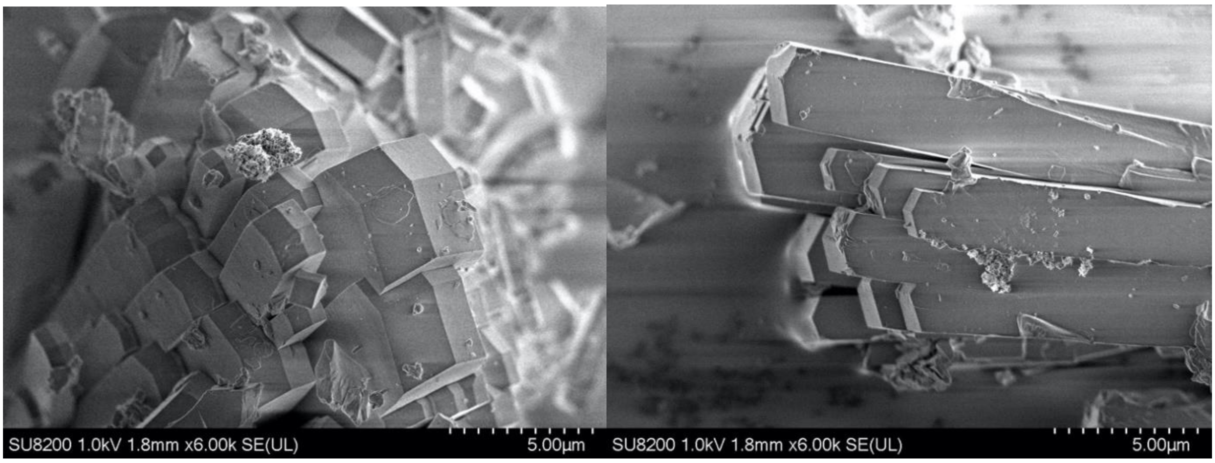

Figure S4. SEM images, from different angles, of Zr_{70}-tI (powder synthesis). The sample consists of bundles of long prismatic crystals. The visible crystal faces are {1 1 0} which constitutes the sides, and the {1 0 1} forming the pyramid-shaped pointy tips.

PXRD

Samples of as-synthesized Zr_{70} samples were mounted as a thin layer of powder on a glass plate-fitted sample holder. Dried powders were measured except for the case of Zr_{70}-tI, where the sample was mounted wet along with some mother liquor. Plastic foil was mounted over the samples to keep them in place and avoid evaporation of crystal water, resulting in broad diffraction signal at approx. 22 and 36 °2θ.

For powder samples of Zr_{70}-tI, both Rietveld and Pawley refinements were performed (using Topas version 5), the results of which are shown in Figure S5. The Pawley refinement was performed in the range of 3.5 to 70 °2θ. The first reflection (1 1 0) had to be excluded from the refinement as it was affected by the beam stopper of the instrument due to the low scattering angle. The refinement shows that the sample has high purity and the unit cell has undergone significant expansion compared to the cell determined at 100 K. The Rietveld refinement was performed in the range of 15 – 70 °2θ,

corresponding to a d-spacing of 6 – 1.43 Å. The region at lower angle is affected by water occupying the pores of the structure, and had to be excluded to obtain a satisfactory refinement. Only unit cell and instrument parameters were allowed to refine freely, and atomic positions were restrained to the same fractional coordinates in the unit cell as determined by SC-XRD. The background was modelled by a Chebychev polynomial with 12 coefficients.

Table S3. Output from Topas Rietveld refinement:

```
Global R-Values


Rexp : 1.19    Rwp : 29.44    Rp  : 20.83  GOF : 24.68
Rexp`: 1.78    Rwp`: 44.00    Rp` : 35.31  DW  : 0.37

Corrections
   Zero error                          0.08056955
   Specimen displacement                    0.004778411
   LP Factor                           27.3
   Surface Roughness Pitschke et al         0.05027686
   Absorption (1/cm)                     75.34618

Structure 1
   Phase name                            Zr70-tI
   R-Bragg                             22.535
   Spacegroup                            I4/mmm
   Scale                               2.42630e-006
   Cell Mass                           31036.699
   Cell Volume (Å^3)                     30159.60780
   Wt% - Rietveld                        100.000
   Double-Voigt|Approach
      Cry size Lorentzian                  639.2
      k:  1  LVol-IB (nm)                  406.953
      k:  0.89  LVol-FWHM (nm)               568.924
   Crystal Linear Absorption Coeff. (1/cm)   127.702
   Crystal Density (g/cm^3)                1.709
   Preferred Orientation (Dir 1 : 1 1 0)     0.1368019
                     (Dir 2 : 0 0 1)     1.660329
      Fraction of Dir 1                    0.1208706
   Lattice parameters
      a (Å)                            40.0102251
      b (Å)                            0.0000000
      c (Å)                            18.8401215
```

Table S4. Output from Topas Pawley refinement:

```
Global R-Values


Rexp : 0.76    Rwp : 16.48    Rp  : 10.24  GOF : 21.72
Rexp`: 0.90    Rwp`: 19.62    Rp` : 12.70  DW  : 0.83

Corrections
   Zero error                          0.07704908
   Specimen displacement                    -0.0001998383
   LP Factor                           27.3

hkl Phase - 1 Pawley method
   Phase name                            hkl_Phase
   R-Bragg                             3.554
   Spacegroup                            I4/mmm
   Cell Mass                           0.000
   Cell Volume (Å^3)                     30204.04594
   Wt% - Rietveld                        0.000
   Double-Voigt|Approach
      Cry size Lorentzian                  540.8
      k:  1  LVol-IB (nm)                  344.307
      k:  0.89  LVol-FWHM (nm)               481.344
   Preferred Orientation (Dir 1 : 0 0 1)     1.615204
                     (Dir 2 : 1 1 0)     0.2143454
      Fraction of Dir 1                    0.9817839
   Lattice parameters
      a (Å)                            40.0337486
      b (Å)                            0.0000000
      c (Å)                            18.8457144
```

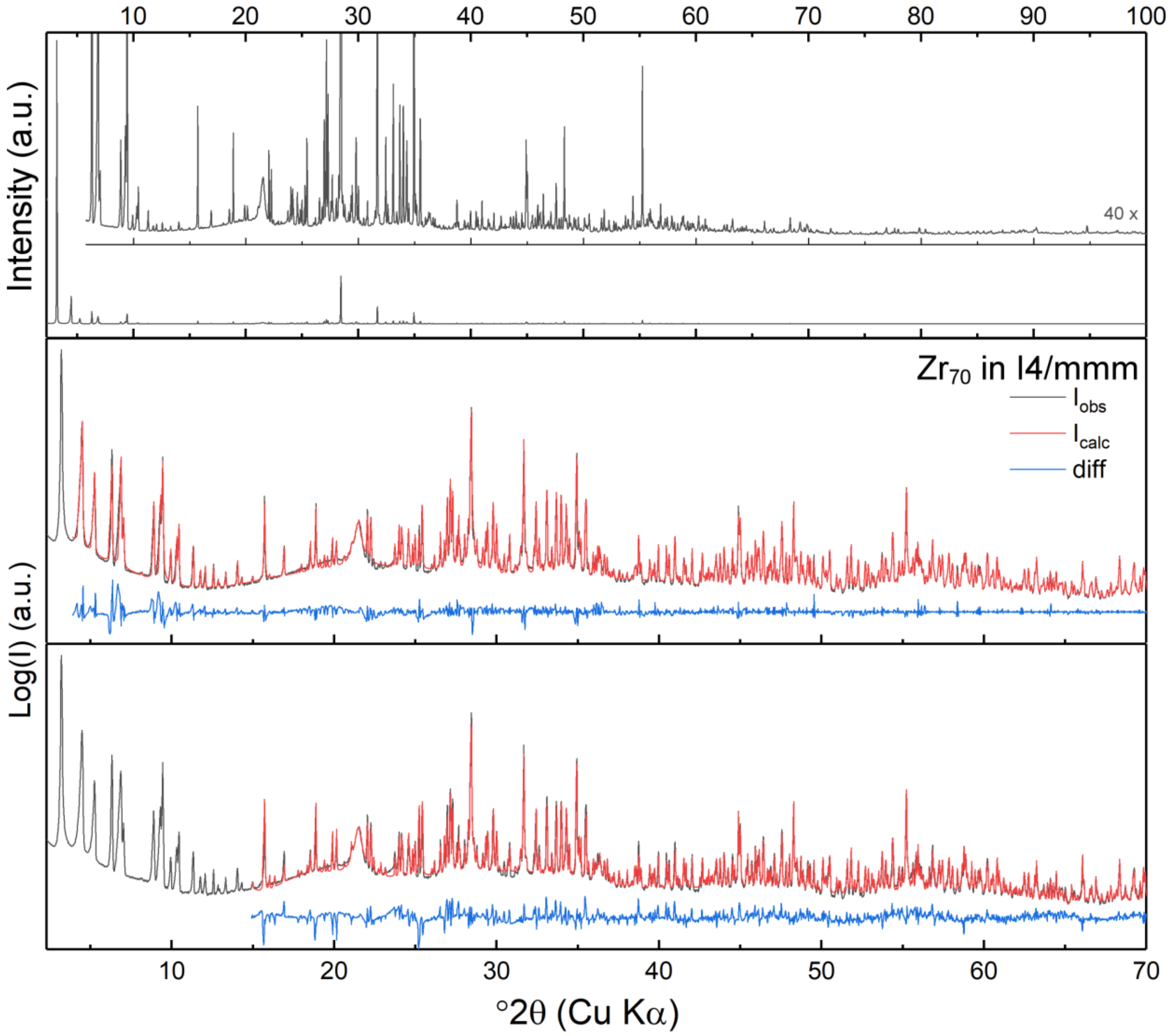

Figure S5. PXRD pattern of Zr_{70}-tI. The intense broad peaks at 22 and 36 °2θ originate from the foil mounted atop the sample. Top: The diffraction pattern shown with a linear y-axis, full pattern and a 40x magnification. Middle: Pawley refinement of Zr_{70}-tI, where the intensity scale is logarithmic. Bottom: Rietveld refinement of Zr_{70}-tI, also with logarithmic intensity scale.

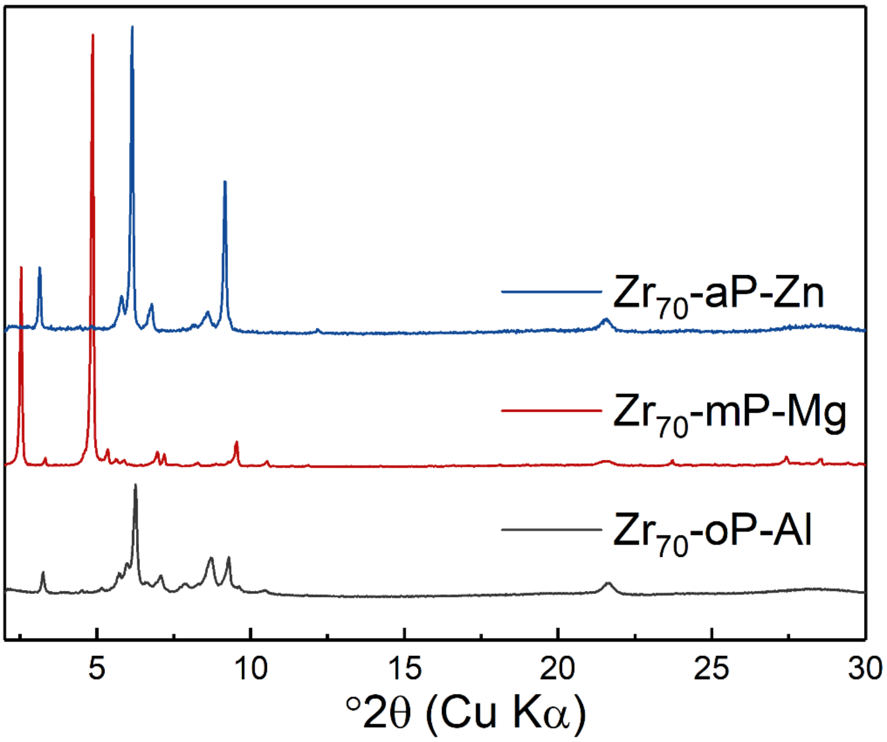

Figure S6. PXRD patterns of Zr_{70}-aP-Zn (top), Zr_{70}-mP-Mg (middle) and Zr_{70}-oP-Al (bottom). The patterns could not be refined due to the low number of visible reflections, but are consistent with large unit cells due to the sharp reflections at low angles.

References

[1] A. Spek, *Acta Crystallogr. Sect. C* **2015**, *71*, 9-18.
[2] Bruker AXS inc., Madison, Wisconsin, USA, 2019.
[3] G. Sheldrick, *Acta Crystallogr. Sect. A* **2015**, *71*, 3-8.
[4] G. M. Sheldrick, *Acta Crystallogr. Sect. A* **2008**, *64*, 112-122.
[5] G. Sheldrick, *Acta Crystallogr. Sect. C* **2015**, *71*, 3-8.
[6] O. V. Dolomanov, L. J. Bourhis, R. J. Gildea, J. A. K. Howard, H. Puschmann, *J. Appl. Crystallogr.* **2009**, *42*, 339-341.
[7] D. A. Gómez-Gualdrón, P. Z. Moghadam, J. T. Hupp, O. K. Farha, R. Q. Snurr, *J. Am. Chem. Soc.* **2016**, *138*, 215-224.

Author Contributions

S.Ø.: Lead data curation (Synthesis, SC-XRD, PXRD, TGA, Adsorption), formal analysis (as previous), investigation, writing of original draft, inc. figures. E.R.: Supporting writing and revision of draft, figures. C.B.: Lead data curation and analysis (TEM). Ø.P.: Data validation (TEM). K.P.L.: Data validation (SC-XRD, TGA, adsorption). U.O.: Lead project administrator and funding acquisition, supporting manuscript revision.
